# Recommendations from the Canadian Association of Head and Neck Surgical Oncology for the Management of Head and Neck Cancers during the COVID-19 pandemic

**DOI:** 10.1186/s40463-020-00448-z

**Published:** 2020-07-29

**Authors:** Daniel A. O’Connell, Hadi Seikaly, Andre Isaac, Justin Pyne, Robert D. Hart, David Goldstein, John Yoo, Canadian Association of Head and Neck Surgical Oncology

**Affiliations:** 1 Canadian Association of Head and Neck Surgical Oncology (CAHNSO), 1E4 8440 112 Street NW, Edmonton, AB T6G 2B7 Canada; 2 Division of Otolaryngology – Head & Neck Surgery, University of Alberta, Edmonton, AB Canada; 3 Division of Otolaryngology, University of Calgary, Calgary, AB Canada; 4 Department of Otolaryngology – Head & Neck Surgery, University of Toronto, Toronto, ON Canada; 5 Department of Otolaryngology – Head & Neck Surgery, University of Western Ontario, London, ON Canada

**Keywords:** COVID-19, SARS-Cov-2, Global pandemic, Head and neck oncology, Head and neck surgery, Clinical practice guideline

## Abstract

**Introduction:**

The SARS-CoV-2 virus (COVID19) pandemic has placed extreme pressures on the Canadian Healthcare system. Many health care regions in Canada have cancelled or limited surgical and non-surgical interventions on patients to preserve healthcare resources for a predicted increase in COVID19 related hospital admissions. Also reduced health interventions may limit the risk of possible transmission of COVID19 to other patients and health care workers during this pandemic. The majority of institutions in Canada have developed their own operational mandates regarding access to surgical resources for patients suffering from Head and Neck Cancers during this pandemic. There is a large degree of individual practitioner judgement in deciding access to care as well as resource allocation during these challenging times. The Canadian Association of Head and Neck Surgical Oncology (CAHNSO) convened a task force to develop a set of guidelines based on the best current available evidence to help Head and Neck Surgical Oncologists and all practitioners involved in the care of these patients to help guide individual practice decisions.

**Main body:**

The majority of head and neck surgical oncology from initial diagnosis and work up to surgical treatment and then follow-up involves aerosol generating medical procedures (AGMPs) which inherently put head and neck surgeons and practitioners at high risk for transmission of COVID19. The aggressive nature of the majority of head and neck cancer negates the ability for deferring surgical treatment for a prolonged period of time. The included guidelines provide recommendations for resource allocation for patients, use of personal protective equipment for practitioners as well as recommendations for modification of practice during the current pandemic.

**Conclusion:**

1. Enhanced triaging should be used to identify patients with aggressive malignancies. These patients should be prioritized to reduce risk of significant disease progression in the reduced resource environment of COVID19 era.
2. Enhanced triaging including aggressive pre-treatment COVID19 testing should be used to identify patients with high risk of COVID19 transmission.
3. Enhanced personal protective equipment (PPE) including N95 masks and full eye protection should be used for any AGMPs performed even in asymptomatic patients.
4. Enhanced PPE including full eye protection, N95 masks and/or powered air purifying respirators (PAPRs) should be used for any AGMPs in symptomatic or presumptive positive COVID 19 patients.

## Introduction

The emergence of the SARS-CoV-2 virus (COVID19) as a novel virus causing severe respiratory illness was first described in December 2019 in Wuhan China. Since that time the World Health Organization (WHO) has declared COVID19 a pandemic (Mar 112,020) and health authorities have been mobilizing to accommodate a predicted surge in patients who require hospital and/or intensive care unit admission due to the direct effects of the virus [1]. The reality of oncologic surgery is that any significant delay in time to treatment will often have a deleterious effect on survival and functional outcomes for the individual patient. This makes managing head and neck cancer patients in the pandemic era much more challenging due to resource limitations placed on the healthcare system by COVID19. In the 2003 Severe Acute Respiratory Syndrome (SARS) pandemic, significant limitations were placed on surgical resource access in Ontario which did have a long term effect both on patient care as well as economic impact on the healthcare system once the pandemic passed and the backlog of surgical patients placed an increased strain on the system resources [2].

Head and neck surgical oncology is particularly challenging in the COVID19 era as all aspects of patient care from initial consultation, to surgical treatment, then post-treatment follow-up involve some degree of AGMPs in standard practice. The upper aerodigestive tract includes the nasal cavities, nasopharynx, oral cavity, oropharynx, hypopharynx, larynx, and trachea. This tract is a zone of intense COVID-19 viral replication [1]. There is growing evidence that personnel who perform procedures in the upper aerodigestive tract, and in particular otolaryngologists Head and Neck Surgeons are at high risk of not only becoming exposed to COVID-19, but also developing severe illness from the virus [3, 4]. In an anecdotal report from Wuhan, it was noted that 14 personnel became may have been infected from a single endoscopic trans-sphenoidal pituitary case, including every OR staff member that was involved in the case [5, 6]. Europe has seen the same high infection rate of otolaryngologists, as reported by intensive care units across the region [7]. There was recently a report of four otolaryngologists in the UK having been infected, two of which are intubated and in critical condition in the ICU, after having contracted COVID-19 from asymptomatic patients [8]. Reliable reports have documented active transmission in asymptomatic cases [6]. The increased risk of contracting COVID-19 in these procedures is thought to be due to the high viral load in the upper respiratory tract [9]. Not only this, but persons who are exposed to high viral loads, such as during surgery as opposed to contracting the virus in the community, are thought to suffer more severe illness due to the release of cytokine storms in these settings [10].

This new and emerging information has led to several specialty societies and health authorities making recommendations on the use of PPE in procedures that are considered aerosol-generating (aerosol generating medical procedures or AGMPs), where standard contact and droplet precautions are not sufficient to protect against aerosolized viral particles. Whereas many of these recommendations vary in the details, all published criteria in the literature now support the use of N95 masks and associated aerosolized droplet precautions with all aerosol-generating medical procedures, regardless of COVID-19 testing. Many guidelines go much farther, recommending PAPRs for all such cases [4, 11]. The question of whether this should be enacted relies on three central issues:

1. The expected rate of community viral burden in the province of practice.
2. The rate of asymptomatic infection.
3. The reliability of COVID-19 testing in asymptomatic patients.

With respect to the first issue, we know that community transmission of COVID-19 is rising across Canada, and is now responsible for more than half of infections in the country [12]. Projections vary widely, but according to the federal health minister, “between 30 and 70 percent of Canadians could become infected with coronavirus” [13]. Countries that did not prepare for this scenario are now facing such consequences, including Italy which is battling a high rate of health worker infections and COVID-related deaths.

With respect to the second issue, emerging data shows that asymptomatic infections may be much more common than previously thought. During the Centers for Disease Control, (CDC) investigation of the Diamond Princess cruise ship outbreak, 46.5% of infected individuals were asymptomatic at the time of testing, and 17.9% of those infected never developed symptoms [14]. This is in alignment with findings in children, which show that more than 15% of patients have asymptomatic infection [15]. There is also ample data to show that active transmission of the virus occurs in asymptomatic persons [16].

Finally, current methods of testing for the COVID-19 virus are not validated for use in asymptomatic persons. Departments of Public Health and the CDC have repeatedly warned about the false negative (and false positive) rate of nasal swabs in asymptomatic patients [16–18]. Although a positive COVID-19 swab in an asymptomatic patient can be useful, a negative swab cannot be relied on to be accurate.

The current COVID19 pandemic makes treating head and neck cancers even more challenging in terms of triaging patients and allocating limited surgical resources. Surgical oncologists worldwide have published recommendations about application and utilization of surgical resources in these challenging times [19–28]. Head and neck surgeons have the difficult task of following chronically ill patients while at the same time mitigating the risk of person to person contact [25, 29]. In doing so, head and neck cancer survivors may be impacted negatively in terms of quality of life as well as disease recurrence detection [23]. Further, those with tracheostomies or laryngeal stomas are at increased risk of transmission and spread of COVID-19 [24].

In summary, we can conclude and/or infer the following from the available data:

1. Personnel who are involved in aerosol-generating medical procedures, in particular otolaryngologists, are at high risk of contracting COVID-19 AND potentially suffering severe or fatal illness from the disease [3, 4, 8, 19, 28, 29].
2. Standard contact and droplet precautions are not sufficient for protecting against COVID-19 in these circumstances [3, 4, 11, 19, 28].
3. There is (and/or soon will be) an increasing burden of the novel coronavirus in the general population [1, 6, 12].
4. A significant proportion of COVID-19 infections are asymptomatic or lack symptoms of upper respiratory tract infection, and these persons can transmit the virus and infect others [16, 17].
5. A negative COVID-19 swab cannot rule out infectivity in asymptomatic persons [16, 17].
6. If you consider an infection rate of only 30% in the general population, with up to 15% of COVID-19 positive patients being asymptomatic, then 1 in 20 patients undergoing surgery may be unknowingly COVID-19 positive, potentially exposing the entire team to high risk of infection without proper precautions.

## Referrals

b. Outpatient, clinic, and office consultations should be limited to urgent or emergent referrals
c. A robust triage referral system should be in place to screen for referrals with prioritization of those with a high risk of malignancy/cancer – these patients would then be deemed urgent referrals
d. Referrals with low likelihood of malignancy or known benign disease should be deferred with records kept for the purposes of later recall where resources and appropriateness allow
e. Virtual / telephone consultation should be considered for all referrals with a risk of malignancy to better ascertain severity and to guide need for face to face consultations.
f. Restrict all patient face to face patient assessments to urgent and emergent interactions.
g. Patients who are at higher risk for adverse outcomes following COVID-19 infection (age > 70 years, known high risk comorbidities, frailty) who meet high risk cancer criteria should be prioritized in a way that minimizes their time in hospital / clinical environments with any elevated risk of COVID 19 exposure

## Diagnostic work-up

h. All patients must undergo clinical screening prior to entering the assessment/treatment area. Minimum screening should include screening questionnaire, travel history, COVID-19 contact risk assessment and body temperature reading. Screening should follow local or regional guidelines where available.
i. Nasopharyngoscopy, rigid nasal endoscopy, flexible laryngoscopy are considered Aerosol Generating Medical Procedures (AGMPs) and appropriate PPE should be worn by providers during these procedures. See - https://www.entcanada.org/wp-content/uploads/NCAL-HN-Oncologic-Surgery-in-COVID-Era_v3.pdf
j. Upper aerodigestive endoscopy should only be performed when absolutely necessary. Staff must not use atomizers for anesthesia and decongestion when performing transnasal endoscopy on patients.
k. COVID negative or unknown status and asymptomatic patients for consultation +/− performing transnasal endoscopy:
  1. The patient should only be scoped once. Only the treating physician should perform the procedure.
  2. PPE should include scrubs, water impermeable gown, N95 mask, face shield or other eye protection, and gloves.
  3. The use of video towers is recommended.
l. COVID positive or presumed positive (symptomatic) patients for consultations +/− performing transnasal endoscopy:
  1. The examination should be performed in room with negative pressure.
  2. Only treating physician or a senior learner should perform the procedure.
  3. PPE should include scrubs, water impermeable gown, surgical hood, full face shield, and double gloves and N95 mask or Powered Air Purifying Respirator (PAPR)
  Appropriate donning and doffing protocols are critical and must be followed when using PPE. An appropriately fit-tested N95 respirator is equally important.
m. Limit diagnostic work up including biopsies, and diagnostic imaging to those cases with a high risk of malignancy
n. Diagnostic imaging requests for patients with a high risk of malignancy should be reduced to the minimum modality(ies) required for safe oncologic treatment decision making.
o. Diagnostic work up including biopsies and diagnostic imaging for cases with low risk of malignancy should be deferred with records kept to initiate work up if required at a later date
p. Ensuring appropriate Personal Protective Equipment (PPE) is available for all health care providers interacting with patients - see https://www.entcanada.org/wp-content/uploads/NCAL-HN-Oncologic-Surgery-in-COVID-Era_v3.pdf

## Multi-disciplinary tumor team (MDT) function

q. MDT / tumor board meetings that require in person interactions that violate social distancing recommendations (< 2 m space between all individuals) should be postponed indefinitely.
r. MDT / tumor board meetings should be converted (where possible) into virtual formats to enable continued function at normal intervals and continued attendance by all required members.

## Surgical management in the COVID-19 era

s. Surgical treatment of Head & Neck Cancer should be prioritized locally to ensure higher risk cases (those with high likelihood of rapid progression, increased risk of death) are receiving priority treatment.
t. As the timeline of duration of the COVID 19 epidemic is unknown, expedition of the surgical management of cases in which a worse outcome is expected if surgery is delayed more than 4–6 weeks including [20, 21]:
  1. Squamous cell carcinoma (SCCA) of the oral cavity, oropharynx, larynx, hypopharynx
  2. Cancers with impending airway compromise
  3. Papillary thyroid cancer with impending airway compromise, rapidly growing, bulky disease
  4. High grade or progressive salivary cancer
  5. T3/T4 melanoma
  6. Rapidly progressing cutaneous SCCA with regional disease
  7. Salvage surgery for recurrent or persistent disease
  8. High grade sinonasal malignancy lacking equivalently efficacious non-surgical treatment
u. PPE during Head & Neck Surgical interventions:
  All recommendations listed below are based on best available current evidence. Further information can be found in the following:
  1. https://www.entcanada.org/wp-content/uploads/NCAL-HN-Oncologic-Surgery-in-COVID-Era_v3.pdf
  2. https://www.entcanada.org/wp-content/uploads/NCAL-HN-Oncologic-Surgery-in-COVID-Era_v3.pdf
  3. https://www.entuk.org/covid-19
  4. http://www.asohns.org.au/about-us/news-and-announcements/latest-news?article=78
  5. https://www.entcanada.org/news-events/covid-19-alerts/
  6. https://www.ncbi.nlm.nih.gov/pmc/articles/PMC7136881/
  7. https://www.baoms.org.uk/_userfiles/pages/files/professionals/covid_19/baoms_baos_covid_advice_update_25_march_2020_final.pdf
v. COVID testing for surgical patients where available is supported by CAHNSO especially for procedures that require manipulation of the upper aerodigestive tract.
  1. Patients that test positive should have their surgery canceled if not urgent or emergent and get rebooked when they test negative.
  2. Patients that test negative should have their surgery booked and PPE precautions should be instituted as listed below (Diagnsotic work- up recommendations 3 a-c), as the limited sensitivity and negative predictive value of testing in asymptomatic patients does not rule out an infectious state.
w. Surgery for COVID unknown or negative patients that requires manipulation of the upper aerodigestive tract:
  1. The operating team should be reduced to essential personnel.
  2. The surgical team PPE should include scrubs, gown, N95 masks, surgical hood, face shield, and double gloves or PAPR, scrubs, gown, and double gloves.
x. Surgery for COVID unknown or negative patients that does not require manipulation of the upper aerodigestive tract.:
  1. PPE as per local or regional health authority recommendations.
y. Surgery for COVID positive patients that includes manipulation of the upper aerodigestive tract.
  1. These cases should be performed on an emergent basis only.
  2. These cases should be performed in a dedicated operating room, following local or regional health authority protocols.
  3. The operating team should be reduced to essential personnel.
  4. The surgical team PPE should include: PAPR scrubs, gown, and double gloves.
  Appropriate donning and doffing protocols are critical and must be followed when using PPE. An appropriately fit-tested N95 respirator is equally important.

## Follow-up

- All non-urgent follow ups should be postponed with records kept for the purposes of later recall where resources and appropriateness allow
- Efforts should be made to maximize the utilization of virtual / telephone follow-ups where appropriate for head and neck cancer follow-ups
- In person patient assessments should be limited to urgent or emergent issues where in person evaluation is required to proceed with treatment decision making.
- All urgent or emergent follow up interactions should follow the same guidelines outlined in sections 2 (referrals) and 3 (diagnostic work-up).

## Clinical trials

dd. Prioritize support for patients currently on clinical trials in active treatment
ee. Consider stopping recruitment into clinical trials where issues arise with patient safety or capacity (resource impact in COVID 19 era)
ff. Local, regional and national guidance should be sought to help manage existing clinical trials for the management of head and neck cancers - https://www.canada.ca/en/health-canada/services/drugs-health-products/drug-products/announcements/management-clinical-trials-during-covid-19-pandemic.html

## Data Availability

Available upon request.
